# The tumour microenvironment of the upper and lower gastrointestinal tract differentially influences dendritic cell maturation

**DOI:** 10.1186/s12885-020-07012-y

**Published:** 2020-06-17

**Authors:** Maria E. Morrissey, Róisín Byrne, Celina Nulty, Niamh H. McCabe, Niamh Lynam-Lennon, Clare T. Butler, Susan Kennedy, Dermot O’Toole, John Larkin, Paul McCormick, Brian Mehigan, Mary-Clare Cathcart, Joanne Lysaght, John V. Reynolds, Elizabeth J. Ryan, Margaret R. Dunne, Jacintha O’Sullivan

**Affiliations:** 1 Department of Surgery, Trinity Translational Medicine Institute, Trinity College Dublin, St James’s Hospital, Dublin 8, Ireland; 2 UCD School of Biomolecular and Biomedical Sciences, UCD Conway Institute, University College Dublin, Belfield, Dublin 4, Ireland; 3 Department of Clinical Medicine, Trinity Translational Medicine Institute, Trinity College Dublin, St. James’s Hospital, Dublin 8, Ireland; 4 GEMS, St James’s Hospital, Dublin, Ireland; 5 Oesophageal Unit, St James’s Hospital, Trinity College Dublin, Dublin, Ireland; 6 Centre for Colorectal Disease, Education and Research Centre, St. Vincent’s University Hospital, Elm Park, Dublin 4, Ireland; 7 Department of Biological Sciences, Health Research Institute, University of Limerick, Castletroy, Co., Limerick, Ireland

**Keywords:** Gastrointestinal cancer, Dendritic cell inhibition, Tumour conditioned media, Tumour microenvironment, Radiotherapy, TNF-α

## Abstract

**Background:**

Only 10–30% of oesophageal and rectal adenocarcinoma patients treated with neoadjuvant chemoradiotherapy have a complete pathological response. Inflammatory and angiogenic mediators in the tumour microenvironment (TME) may enable evasion of anti-tumour immune responses.

**Methods:**

The TME influence on infiltrating dendritic cells (DCs) was modelled by treating immature monocyte-derived DCs with Tumour Conditioned Media (TCM) from distinct gastrointestinal sites, prior to LPS-induced maturation.

**Results:**

Cell line conditioned media from gastrointestinal cell lines inhibited LPS-induced DC markers and TNF-α secretion. TCM generated from human tumour biopsies from oesophageal, rectal and colonic adenocarcinoma induced different effects on LPS-induced DC markers - CD54, CD80, HLA-DR, CD86 and CD83 were enhanced by oesophageal cancer; CD80, CD86 and CD83 were enhanced by rectal cancer, whereas CD54, HLA-DR, CD86, CD83 and PD-L1 were inhibited by colonic cancer. Notably, TCM from all GI cancer types inhibited TNF-α secretion. Additionally, TCM from irradiated biopsies inhibited DC markers. Profiling the TCM showed that IL-2 levels positively correlated with maturation marker CD54, while Ang-2 and bFGF levels negatively correlated with CD54.

**Conclusion:**

This study identifies that there are differences in DC maturational capacity induced by the TME of distinct gastrointestinal cancers. This could potentially have implications for anti-tumour immunity and response to radiotherapy.

## Background

The 5-year overall survival rates across gastrointestinal (GI) cancer types vary, with oesophageal adenocarcinoma (OAC) low at 18–19%, whereas colonic and rectal adenocarcinoma rates stand at 58–59% according to the National Cancer Registry Ireland [1]. OAC rates have increased by almost half in recent years in Western countries, mirroring the increase in obesity [2]. Colorectal cancer (CRC) is the third most common cancer worldwide with rectal cancer accounting for approximately 35% of CRC cases. Typically, epidemiological and scientific studies group colon and rectal cancer together, despite their different standard treatment regimens [3, 4]. Standard treatment for oesophageal and rectal adenocarcinoma involves neoadjuvant chemoradiotherapy (CRT) to shrink the tumour prior to surgical resection, whereas for colonic adenocarcinoma the standard treatment involves surgical resection followed by adjuvant targeted therapies [5–7]. Response to CRT is highly variable with just 10–30% of patients achieving a complete pathological response, which is linked with higher 5-year survival rates, for both oesophageal and rectal cancer [8–13]. Tumours have different levels of radiosensitivity and a number of cellular processes and immune mechanisms have been implicated in radioresponse phenotypes [14–16]. Understanding the key components of the immune system which are modulated by the tumour microenvironment (TME) may offer insights into ways to improve the clinical outcome for patients with GI cancers by identifying either prognostic biomarkers or novel therapeutic strategies.

Dendritic cells (DCs) are professional antigen-presentation cells responsible for activation of T cells and thus orchestration of the adaptive immune response [17]. Immature DCs recognise and capture antigens and are characterised by low expression of maturation and co-stimulatory markers such as CD83, CD54, CD80, CD86; HLA-DR for antigen presentation and other immunoinhibitory markers such as PD-L1 [18, 19]. DC maturation, a crucial factor for efficient T cell activation, is triggered in response to various inflammatory mediators and TLR-dependent activation, such as bacterial LPS via TLR4, leading to the increased expression of several cell surface markers by DCs, migration to lymph nodes and presentation of antigens via MHC class I and II molecules to activate CD4^+^ and CD8^+^ T cells [20]. Factors such as IL-10 and VEGF in the TME influence DC function and these can inhibit IL-12p70 and TNF-α production from DCs [21, 22]. DCs which secrete high levels of IL-12p70 induce anti-tumour immunity, as they have increased capacity to enhance natural killer cell activity, skew T cell responses to T helper (Th)-1 type and prime tumour antigen specific T cells [23, 24]. Decreased IL-12p70 expression is associated with suppressed endocytic activity and antigen-presentation machinery, and also decreased motility of anti-tumour immune cells to the tumour site [25]. TNF-α released by immunostimulatory DCs can also act to enhance T cell stimulatory capacity, while increasing IL-12 production from DCs and decreasing production of the immunosuppressive cytokine IL-10 [26, 27].

Known risk factors for the development of GI cancers include inflammatory disorders, specifically Barrett’s oesophagus for OAC and inflammatory bowel disease for CRC [28–30]. Not only is inflammation a hallmark of cancer, it plays a pivotal role in modulating radiation responsiveness of tumours [31]. Radiation can elicit the systemic release of the TLR ligands, damage-associated molecular patterns (DAMPs), after oesophageal irradiation or locally after targeted tumour irradiation, such as treatment for CRC [32–37]. TLR-dependent activation of DCs after irradiation supports the use of low dose hypofractionated radiotherapy as an adjuvant to immunotherapy to enhance its effect, however either very low or high levels may be immunosuppressive [32]. Direct immunomodulatory effects of irradiation on immune cells have been reported, such as altered IL-12 production from DCs [33, 34]. It is important to understand the immunosuppressive nature of the TME for tumour-infiltrating DCs, which may limit the success of different treatments, e.g. immunotherapies, including DC vaccines [38].

We have previously described the immunosuppressive effect of the colonic TME which inhibits LPS-induced DC maturation [18, 20, 39–41]. Using a similar experimental outline as we described previously, in this study we investigated the effects on DC maturational capacity across different human GI cancers, using conditioned media from cell lines (in vitro conditioned media) and treatment-naïve tumour biopsies (ex vivo TCM) (Supplementary Fig. 1 A). While in vitro conditioned media represents the secretome from cancer epithelial cells, ex vivo conditioned media is a more complex model containing the soluble contributions from many different cells within the tumour microenvironment [18, 20, 39–41]. Due to our interest in understanding the tumour microenvironment in both upper and lower GI tract cancers; oesophageal, rectal and colonic adenocarcinoma were investigated. This study describes for the first time that there were unexpected differences induced by the TCM on maturation of monocyte-derived DCs. Here, oesophageal cancer induced the highest level of DC maturation markers, rectal cancer induced moderate levels of DC maturation markers and colonic cancer significantly inhibited DC maturation markers. Interestingly for all GI cancer types examined here, in vitro and ex vivo TCM significantly inhibited TNF-α secretion from DCs. In addition, we modelled radiotherapy of oesophageal and rectal biopsies and found that TCM from 2Gy-irradiated tumours inhibited LPS-induced DC markers. Differential levels of specific inflammatory and angiogenic mediators were detectable in ex vivo TCM of GI cancers that correlated with DC maturation.

## Methods

### Cell culture and irradiation

Human oesophageal adenocarcinoma cell lines - isogenic OE33 parental (OE33 P) and radioresistant (OE33 R) lines [42], and commercially available colorectal adenocarcinoma - SW480 and SW620 lines, were maintained in RPMI-1640 medium (Invitrogen) supplemented with 10% v/v FBS and 1% v/v penicillin/streptomycin in a humidified atmosphere with 5% CO_2_ at 37 °C. 3 × 10^5^ cells in 2 ml media were seeded in 6-well plates, allowed adhere overnight and were 0Gy- (mock) or 2Gy-irradiated at 70% confluence using an X-Ray generator (RS 225 system, using X-rays from a Tungsten target at a rate of 3.25 Gy/min) (Gulmay Medical, UK). Following 24-h culture, the supernatant was harvested and frozen as in vitro conditioned media from three independent biological replicates.

### Human ex vivo tumour explant culture and irradiation

All tissue was obtained with prior written informed consent from each patient, and ethical approval was granted by the Adelaide and Meath Hospital (AMNCH), Tallaght, Dublin Research Ethics Committee. All biopsy samples used were treatment-naïve (Supplementary Table 1). Patient-matched tumour biopsies taken from patients with oesophageal or rectal adenocarcinoma were either 0Gy- (mock) or 2Gy-irradiated using an X-Ray generator (RS 225 system, using X-rays from a Tungsten target at a rate of 3.25 Gy/min) (Gulmay Medical, UK). For oesophageal adenocarcinoma, fresh tissue was cultured for 24 h in complete M199 (cM199) – M199 media (Invitrogen) media supplemented with 10% v/v FBS, 1% v/v penicillin/streptomycin and 1 μg/ml insulin (Sigma-Aldrich UK). For rectal adenocarcinoma samples, fresh tissue was washed and cultured for 48 h in complete RPMI (cRPMI) - RPMI-1640 (Invitrogen) media supplemented with 10% FBS, 1% penicillin/streptomycin, 1% fungizone and 50 μg/ml gentamicin. For colonic adenocarcinoma, the tissue was cultured for 72 h in cRPMI - RPMI-1640 media (Invitrogen) supplemented with 10% FBS, 1% penicillin/streptomycin and 1% fungizone. Culturing was performed in a humidified atmosphere with 5% CO_2_ at 37 °C. Ex vivo TCM was harvested and frozen at − 80 °C.

### DC isolation and culture

Human monocyte-derived immature DCs were generated from peripheral blood mononuclear cells (PBMCs) obtained from buffy coat preparations (National Blood Centre, St. James’s Hospital, Dublin) by density gradient centrifugation (Lymphoprep) as we previously described (Supplementary Fig. 1 A) [18, 43]. Briefly, monocytes were isolated by positive selection using anti-CD14 magnetic microbeads as described by the manufacturer (Miltenyi Biotec) and seeded at a density of 1 × 10^6^ cells/mL in 6-well plates in 3 mL of RPMI-1640 medium containing 10% defined HyClone FBS (Thermo Scientific), 1% penicillin/streptomycin, 1% fungizone, human granulocyte macrophage colony-stimulating factor (50 ng/mL; Immunotools), and human IL-4 (70 ng/mL; Immunotools) in a humidified atmosphere with 5% CO_2_ at 37 °C. Cells were fed at day 3 by replacing half the medium made up with fresh cytokines as above. At day 6, CD11c^+^ cells exhibited an immature DC phenotype capable of upregulating cell surface markers following LPS activation.

### Stimulation of monocyte-derived DCs

Freshly generated DCs were plated in 96-well plates at 2 × 10^5^ cells in 200 μL RPMI-1640 media supplemented with 10% defined Hyclone FBS (Thermo Fisher Scientific) and stimulated with a 1:2 dilution of conditioned media, or matched background media controls, for 4–5 h before exposure to 10 μg ml-1 of ultrapure TLR4 agonist *Escherichia coli* lipopolysaccharide (LPS-EB; Invivogen) overnight. Supernatants were harvested and frozen for ELISA analysis, and cells were assessed for expression of surface markers by flow cytometry as we described previously (Supplementary Fig. 1 A) [18, 20, 39–41].

### Flow cytometry

DCs were stained with the following antibody panel: phycoerythrin (PE)- anti-CD80 (2D10), PerCP-Cy5.5- anti-CD86 (IT2.2), Pe-Cy7- anti-CD83 (HB15), Brilliant Violet 421- anti-PD-L1 (29E.2A3), Brilliant Violet 510- anti-CD11c (3.9), allophycocyanin (APC)- anti-CD54 (HA58), and APC-Cy7- anti-HLA-DR (L243) (Biolegend). DC preparations were acquired on DAKO CyAn ADP flow cytometer (Beckman Coulter) with compensation performed with positive and negative antibody capture beads (BD Biosciences). Gating on and analysis of CD11c + cells was performed using FlowJo software (Tree Star Inc.) to determine Mean Fluorescence Intensity (MFI). The gating strategy and Fluorescence Minus One (FMO) staining controls are shown (Supplementary Fig. 1 B-C) and representative histograms are graphed (Supplementary Fig. 2).

### Elisa

Levels of IL-12p70 and TNF-α in DC supernatant were quantified by DuoSet sandwich Enzyme-Linked Immunosorbent Assay (ELISA) kits according to the manufacturer’s protocol (R&D Systems). Multiplex ELISA was performed to simultaneously assess levels of ten inflammatory markers (IL-2, MMP2, MMP9, CCL2, IL-6, CCL20, TNF-α, IL-1β and IL-10) or seven angiogenic markers (ICAM-1, VCAM-1, bFGF, VEGF, PAI1 and Ang-2) in ex vivo TCM, according to the manufacturer’s protocol (Meso Scale Diagnostics) [44].

### Data analysis

Data displayed in graphs is from one healthy PBMC donor for in vitro TCM (*n* = 3). Data displayed in graphs is from one healthy PBMC donor per cancer type for ex vivo TCM with *n* = 7–14 tumour samples as indicated in the legends. Statistical analyses were carried out using GraphPad Prism v5 for Windows (GraphPad software). Paired or unpaired t-test, or ANOVA with post hoc Dunnett’s t-tests were used to compare groups as indicated. A *p* value of less than 0.05 was considered to be significant in all of the analyses where * *p* ≤ 0.05; ** *p* ≤ 0.01 and *** *p* ≤ 0.001 for t-tests.

## Results

### Differential DC maturation induced by conditioned media from OAC and CRC cell lines, while both inhibited TNF-α secretion

**Fig. 1:**
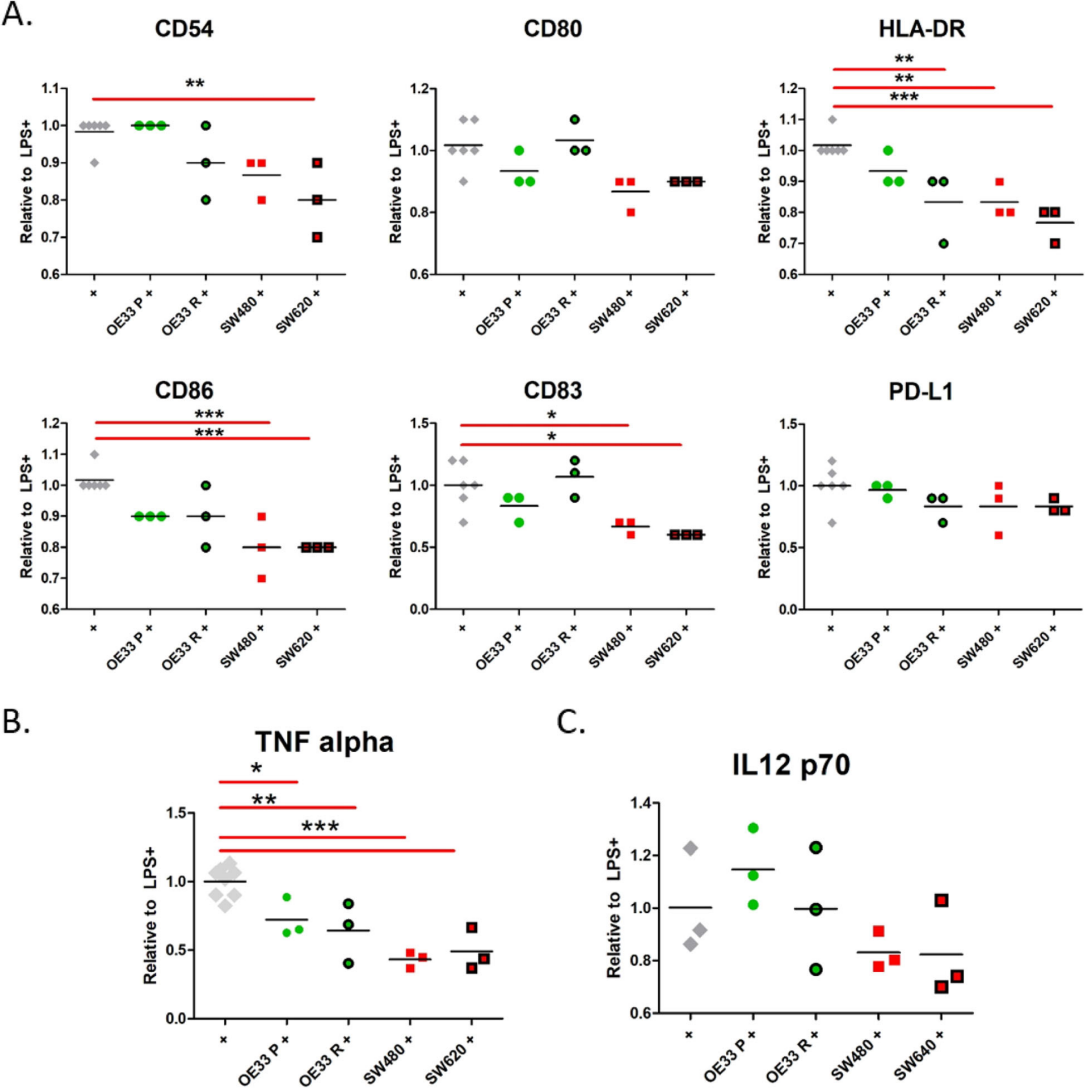
in vitro conditioned media from gastrointestinal cell lines influenced DC maturation. **a-c** Data from DC surface flow cytometry (**a**) and ELISA of DC supernatants (**b-c**) was produced from one healthy PBMC donor pre-treated with supernatants of three independent biological replicates of conditioned media harvested from OAC cell lines (green circles) and CRC cell lines (red squares). **a** HLA-DR was inhibited by conditioned media from the OAC cell line, OE33 Radioresistant, relative to LPS-induced levels (+, grey diamonds), whereas there was inhibition of five DC markers (CD54, CD80, HLA-DR, CD86 and CD83) by conditioned media from one or both of the CRC cell lines. **b-c** LPS-induced levels of TNF-α was inhibited by OAC and CRC conditioned media (**b**), though no significant differences were observed for IL-12 p70 (**c**). DC maturation levels are shown relative to LPS-induced levels in background media (+, grey diamonds) and statistical comparison of in vitro conditioned media+LPS (*n* = 3) is performed relative to LPS-induction alone (+, n = 3–6). Statistically significant (ANOVA with Dunnett’s Multiple Comparison Test) inhibition of DC maturation relative to the LPS-induced levels (+, grey diamonds) is indicated by asterisks

We investigated if the maturational capacity of DCs could be influenced by conditioned media harvested from cell lines from upper and lower GI tract cancers. We found that in vitro conditioned media from three independent biological replicates of OAC and CRC cell lines modulated LPS-induced DC maturation (Fig. 1). TCM from the OE33 Radioresistant (OE33 R) cells significantly reduced HLA-DR expression following LPS-treatment. Whereas the TCM from both CRC lines - SW480 and SW620 inhibited multiple DC surface markers relative to LPS in background media (+) (Fig. 1a). In vitro conditioned media from both SW480 and SW620 lines inhibited HLA-DR, CD86 and CD83 and additionally, SW480 inhibited CD80 and SW620 inhibited CD54. The effect of in vitro conditioned media on IL-12p70 and TNF-α production by DCs was also examined relative to levels induced by LPS in background media (+) (Fig. 1b-c). The conditioned media of both OAC and CRC lines significantly inhibited LPS-induced TNF-α in DC supernatants (Fig. 1b). No significant changes were observed for LPS-induced IL-12p70 in DC supernatants (Fig. 1c).

In summary, the TCM from OAC cell lines inhibited only HLA-DR on DCs and it inhibited levels of soluble TNF-α, whereas the TCM from CRC cell lines inhibited five DC surface markers in addition to soluble TNF-α.

### TCM from human tumour biopsies induced different effects on DC maturation based on cancer type

**Fig. 2:**
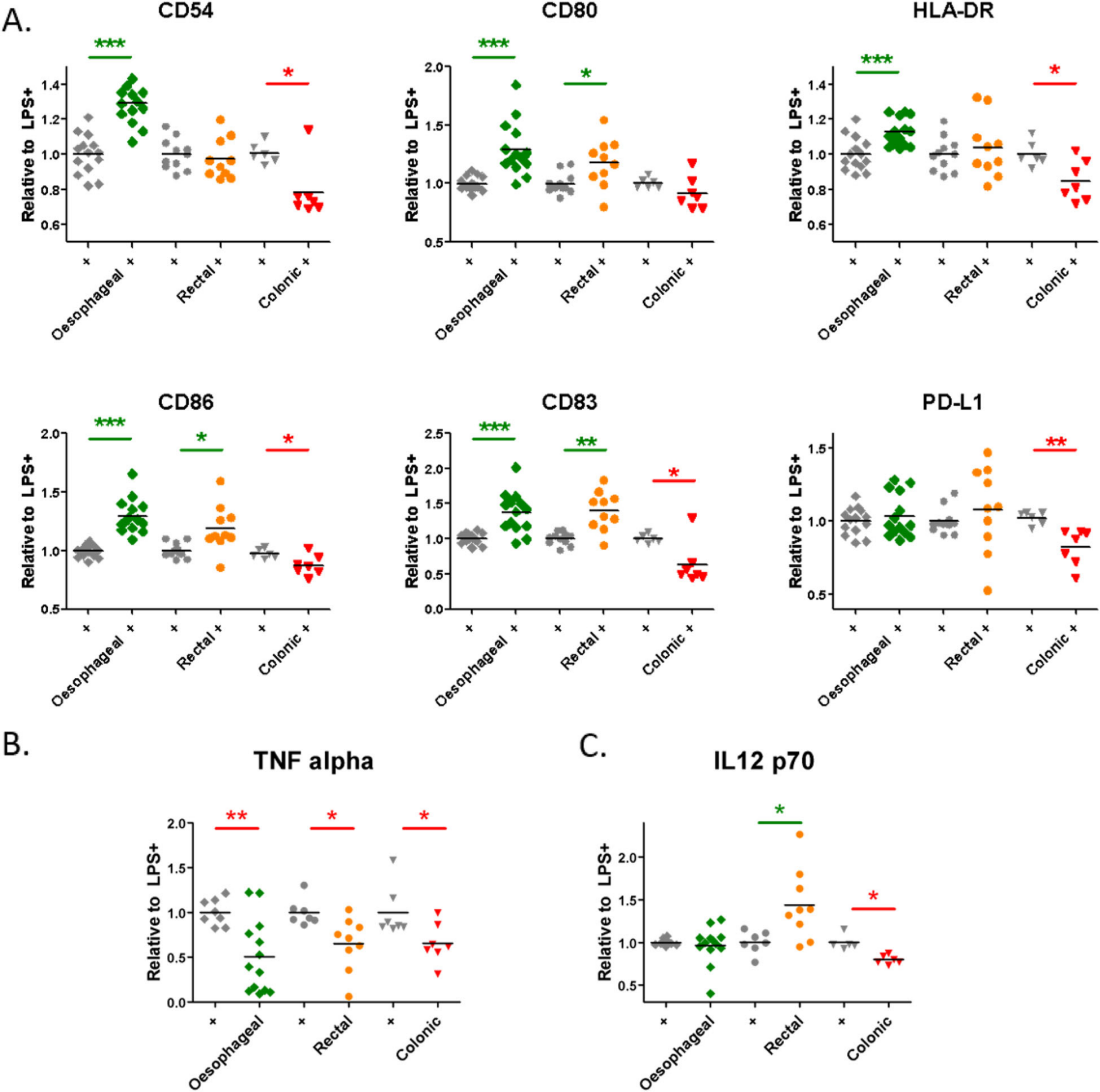
ex vivo TCM from distinct gastrointestinal adenocarcinoma types induced differential effects on DC maturation. **a-c** Data from DC surface flow cytometry (**a**) and ELISA of DC supernatants (**b-c**) was produced from one healthy PBMC donor per cancer type pre-treated with conditioned media from treatment-naïve biopsies of oesophageal (green squares, *n* = 14), rectal (orange circles, *n* = 10) and colonic (red triangles, *n* = 7) adenocarcinoma. **a** Oesophageal TCM enhanced five markers (CD54, CD80, HLA-DR, CD86 and CD83), rectal TCM enhanced three markers (CD80, CD86 and CD83) and colonic TCM inhibited five markers (CD54, HLA-DR, CD86, CD83 and PD-L1) over LPS-induced levels (+, where grey diamonds, circles and triangles indicate LPS-induced levels in appropriate background media for oesophageal, rectal and colonic adenocarcinoma TCM respectively). **b-c** There was inhibition of TNF-α (**b**) by conditioning with oesophageal, rectal or colonic TCM, whereas only colonic TCM inhibited IL-12 p70 (C). DC maturation levels are shown relative to LPS-induced maturation (+, grey bar) and statistical comparison of ex vivo TCM + LPS (n = 3) is performed relative to LPS-induction alone (+). Statistically significant (unpaired t-test) modulation of DC maturation relative to LPS control (+, grey bars to the left of each cancer type) is indicated by asterisks, where green asterisks indicate significant enhancement and red asterisks indicates significant inhibition and * *p* ≤ 0.05; ** *p* ≤ 0.01 and *** *p* ≤ 0.001

We investigated if the maturational capacity of DCs is influenced by three distinct GI tract cancers. We found that ex vivo TCM from treatment-naïve oesophageal, rectal and colonic adenocarcinoma tissues modulated LPS-induced DC maturation (Fig. 2, Supplementary Fig. 2). TCM from oesophageal adenocarcinoma significantly enhanced CD54 (*p* < 0.001), CD80 (*p* < 0.001), HLA-DR (*p* = 0.001), CD86 (*p* < 0.001) and CD83 (*p* < 0.001) compared to LPS-induction in background media alone, cM199 (+) (Fig. 2a). Moreover, the TCM from rectal adenocarcinoma significantly enhanced CD80 (*p* = 0.028), CD86 (*p* = 0.016) and CD83 (*p* = 0.002) compared to LPS-induction in background media alone, cRPMI (+) (Fig. 2a). Whereas, the TCM from colonic adenocarcinoma significantly inhibited CD54 (*p* = 0.011), HLA-DR (*p* = 0.013), CD86 (*p* = 0.021), CD83 (*p* = 0.018) and PD-L1 (*p* = 0.006) compared to LPS-induction in background media alone, cRPMI (+). Similar findings were observed in the unstimulated setting, without LPS, where with both oesophageal and rectal TCM, all DC markers were enhanced relative to unstimulated DCs, whereas with colonic TCM, DC markers were at a similar level to unstimulated DCs (Supplementary Fig. 3).

The effect of ex vivo TCM on LPS-induced levels of IL-12p70 and TNF-α in DC supernatants were also examined (Fig. 2b-c). The TCM of oesophageal, rectal and colonic adenocarcinoma significantly inhibited levels of TNF-α in DC supernatants (*p* = 0.004, *p* = 0.013 and *p* = 0.026 respectively) (Fig. 2b). Whereas for IL-12p70, while oesophageal TCM had no effect on LPS-induced levels, rectal TCM significantly enhanced IL-12p70 levels (*p* = 0.017) and colonic TCM significantly inhibited IL-12 p70 levels (*p* = 0.001) compared to LPS-induced levels (Fig. 2c).

Despite some inter-individual variability, ex vivo TCM from oesophageal, rectal and colonic adenocarcinoma differentially primed immature DCs to respond to subsequent LPS stimulation. There were no significant correlations with clinicopathological parameters examined (Supplementary Table 1).

### TCM of 2Gy-irradiated TME from GI cancers significantly inhibited LPS-induced DC markers

As the standard-of-care treatment for oesophageal and rectal cancer includes radiotherapy which influences the immune system through unclear mechanisms, we investigated if the maturational capacity of DCs is influenced by irradiated GI tract cancers*.* We found that conditioned media harvested from irradiated OAC lines further inhibited LPS-induced DC maturational capacity. While only HLA-DR was significantly inhibited by conditioned media from 0Gy-irradiated OE33 R line compared to LPS-induced levels, CD86, CD80 and PD-L1 were also inhibited by conditioned media from 2Gy-irradiated OAC lines (Fig. 1, Supplementary Fig. 4). While most DC markers were significantly inhibited by in vitro conditioned media of 0Gy-irradiated CRC lines, no additional effect was induced by the 2Gy-irradiated CRC lines (Fig. 1, Supplementary Fig. 4).

**Fig. 3:**
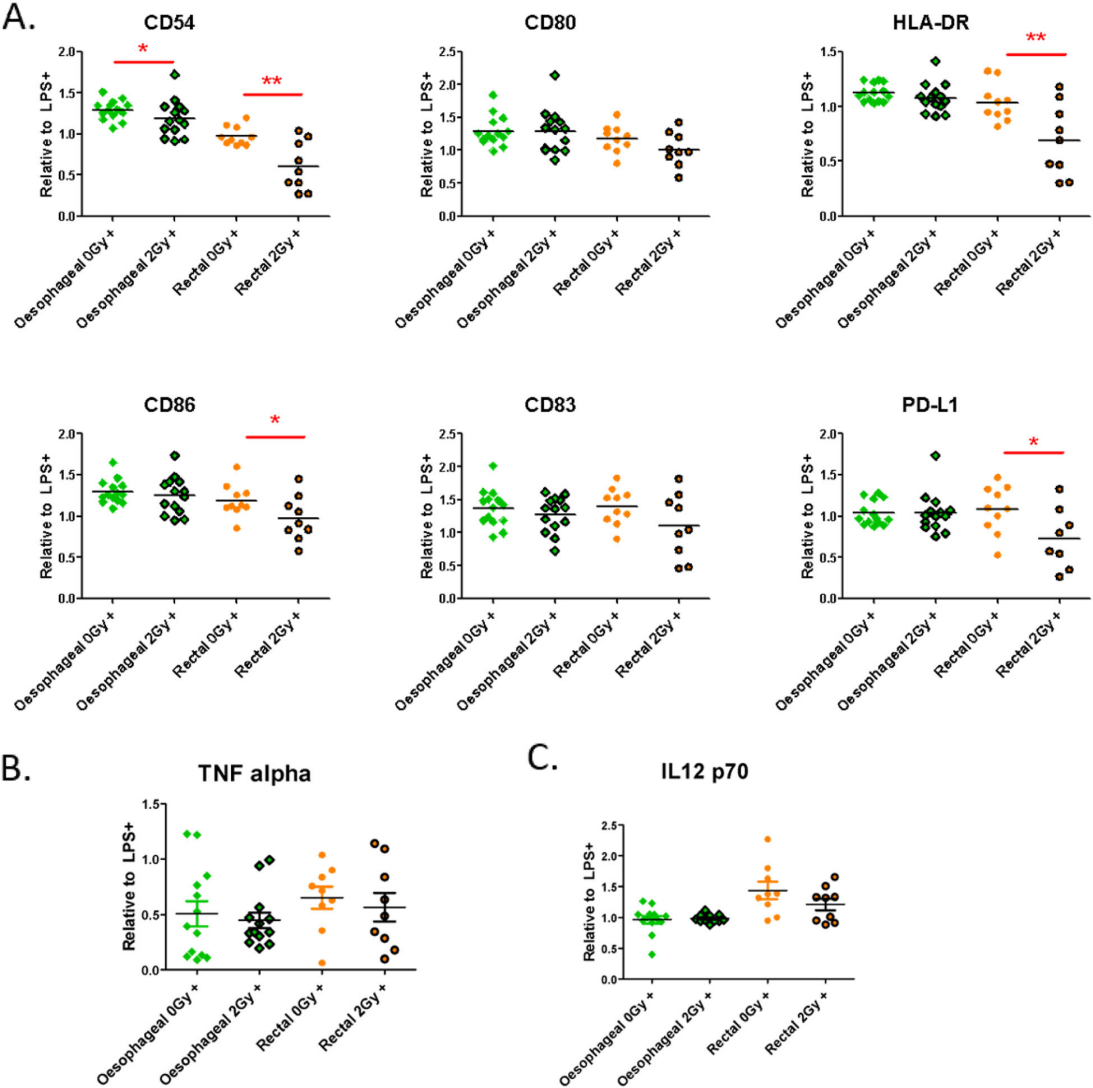
ex vivo TCM of 2Gy-irradiated gastrointestinal adenocarcinoma significantly inhibited DC markers. **a-c** Data from DC surface flow cytometry (**a**) and ELISA of DC supernatants (**b-c**) was produced from one healthy PBMC donor per cancer type pre-treated with conditioned media from 0Gy and 2Gy-irradiated patient-matched biopsies of oesophageal (green squares, *n* = 14), and rectal (orange circles, n = 10) adenocarcinoma. **a** Patient-matched ex vivo TCM from 0Gy- (no borders) or 2Gy (black borders)-irradiated biopsies of oesophageal and rectal adenocarcinoma altered LPS-induced DC maturation. CD54 was inhibited by 2Gy-irradiated oesophageal TCM relative to mock irradiation. CD54, HLA-DR, CD86 and PD-L1 were inhibited by 2Gy-irradiated rectal TCM relative to mock irradiation. **b-c** There was no effect of the 2Gy-irradiated TME on TNF-α (**b**) or IL-12 p70 (**c**) levels. Statistically significant (paired t-test) modulation of DC maturation between 0Gy- and 2Gy-irradiated patient-matched TCMs is indicated by asterisks, where red asterisks indicate significant inhibition and * *p* ≤ 0.05; ** *p* ≤ 0.01 and *** *p* ≤ 0.001

Similarly, we found that ex vivo TCM generated from biopsies of oesophageal or rectal adenocarcinoma, that received 0Gy- (mock) or 2Gy-irradiation, differentially altered DC maturational capacity (Fig. 3, Supplementary Fig. 5). In the LPS-stimulated setting, TCM from 2Gy irradiated oesophageal adenocarcinoma biopsies inhibited levels of CD54, compared to 0Gy patient-matched biopsies (*p* = 0.024), whereas, the levels of CD80, HLA-DR, CD86 and PD-L1 were not differentially altered (Fig. 3a). TCM from 2Gy irradiated rectal adenocarcinoma significantly inhibited LPS-induced DC maturation, compared to 0Gy patient-matched biopsies for CD54 (*p* = 0.003), HLA-DR (*p* = 0.007), CD86 (*p* = 0.050) and PD-L1 (*p* = 0.036) (Fig. 3a). There were no differences in LPS-induced levels of TNF-α or IL-12p70 in DC supernatants between 0Gy- and 2Gy-irradiated biopsies for either oesophageal or rectal adenocarcinoma (Fig. 3b-c).

### Differential levels of inflammatory and angiogenic mediators in the ex vivo TCM of oesophageal, rectal and colonic adenocarcinoma biopsies correlated with DC maturation marker CD54

**Fig. 4:**
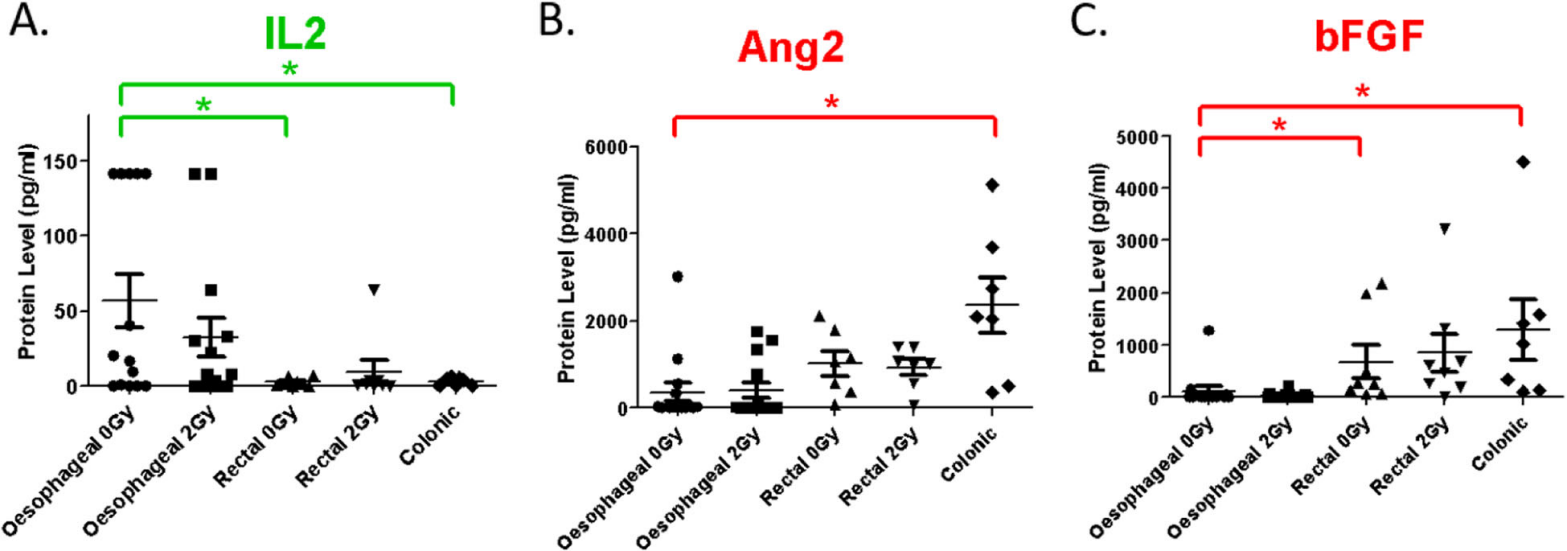
Differential levels of inflammatory and angiogenic mediators in ex vivo TCM of oesophageal, rectal and colonic adenocarcinoma tumour biopsies. Ex vivo TCM contained varying raw levels of inflammatory and angiogenic mediators. IL-2 was at significantly higher levels in oesophageal than rectal and colonic adenocarcinoma TCM (green asterisks). Ang-2 was at significantly lower levels in oesophageal than colonic TCM (red asterisks). bFGF was at significantly lower levels in oesophageal than rectal and colonic TCM (red asterisks). Statistically significant (unpaired t-test) levels of secreted mediators in mock or unirradiated TCM of oesophageal (n = 14), rectal (*n* = 8) or colonic (*n* = 7) biopsies is indicated by asterisks, where green asterisks indicate higher levels in the oesophageal TCM than in rectal or colonic TCM and red asterisks indicate lower levels, * *p* ≤ 0.05. No significant differences were observed between ex vivo TCM from patient-matched 0Gy- and 2Gy-biopsies

As inflammatory and angiogenic mediators influence DCs, we investigated if levels of specific inflammatory and angiogenic mediators secreted by distinct GI tract cancers correlated with DC inhibition. Ex vivo TCM of oesophageal (*n* = 14), rectal (*n* = 8) and colonic (*n* = 7) human tumour biopsies were screened simultaneously for raw levels of ten inflammatory and seven angiogenic markers. We found that IL-2 was at significantly higher levels in oesophageal than rectal and colonic adenocarcinoma TCM, Ang-2 was at significantly lower levels in oesophageal than colonic TCM and bFGF was at significantly lower levels in oesophageal than rectal and colonic TCM (Fig. 4). Indeed, levels of IL-2, Ang-2 and bFGF across oesophageal, rectal and colonic adenocarcinoma correlated with LPS-induced levels of the DC surface marker CD54. Specifically, IL-2 had a Pearson correlation of r = 0.3 (*p* = 0.010), Ang-2 had a Pearson correlation of r = − 0.3 (*p* = 0.010) and bFGF had a Pearson correlation of r = − 0.3 (*p* = 0.022). The other DC markers - CD80, HLA-DR, CD86, CD83 and PD-L1, did not show any significant correlations across cancer types (data not shown). Other inflammatory (MMP2, MMP9, CCL2, IL-6, CCL20, TNF-α, IL-1β and IL-10) and angiogenic (ICAM-1, VCAM-1, VEGF and PAI1) mediators did not show significant correlations with DC inhibition (data not shown). There were no differences in levels of any of the inflammatory or angiogenic mediators examined between 0Gy- and 2Gy-irradiated patient-matched TCMs for either oesophageal or rectal adenocarcinoma biopsies (Fig. 4a-c).

## Discussion

The aim of this study was to examine if DC maturation was influenced by three distinct cancers of the GI tract - oesophageal, rectal and colonic adenocarcinoma. As radiotherapy induces unclear effects in terms of immunomodulation, we also investigated the effect of radiotherapy in this setting. The influence of the gastrointestinal TME on infiltrating DCs was modelled by conditioning immature monocyte-derived DCs with TCM, followed by subsequent LPS maturation, to investigate the effect of the TME on the maturational capacity of DCs as previously described [18]. The effect of TCM on LPS-induced levels of DC markers CD54, CD80, HLA-DR, CD86, CD83 and PD-L1, and two secreted cytokines IL-12p70 and TNF-α in DC supernatants, as indicators for DC maturation were examined (Supplementary Fig. 1 A). We describe the levels of DC maturational capacity induced by conditioned media from oesophageal and colorectal cell lines. While TCM from OE33 oesophageal cell lines inhibited levels of HLA-DR only, TCM from CRC lines inhibited five DC markers - CD54, CD80, HLA-DR, CD86 and CD83. These markers are upregulated on the surface of DCs in order to ensure DCs can stimulate an effective T cell response [18, 19]. CD83 is the most prominent surface marker for fully matured human DCs and enhances DCs’ T cell stimulatory capacity [45]. CD80 and CD86 are also co-stimulatory and engage T cells, CD54 promotes DC-T cell binding and HLA-DR, otherwise known as MHC class II, allows for antigen presentation to CD4+ T cells [45–47]. Altered HLA class II cell surface expression, a mechanism by which tumour cells escape from T cell responses, has been reported in many types of cancer [48]. Normally antigen presentation cells, including DCs, constitutively express HLA class II molecules on the cell membrane, while only minor percentage of tumours and tumour cells express HLA-DR. In this study, we found opposing results for HLA-DR for oesophageal cancer, where the in vitro model inhibited HLA-DR on DCs and the ex vivo model enhanced HLA-DR on DCs. The reason for the modulation of HLA-DR in oesophageal cancer is unclear. This finding could potentially indicate that the tumour epithelial cells may contribute to the inhibition of HLA-DR, rather than the more complex oesophageal tumour microenvironment which contains both epithelial and non-epithelial cells. Whereas we found that both in vitro and ex vivo models of colonic cancer inhibited HLA-DR. These are contradictory findings and the reasons are unclear, in particular because we have previously shown that HLA-DR expression in tumour epithelium is an independent prognostic indicator in oesophageal adenocarcinoma patients, and speculate that for patients with enhanced survival, tumour epithelial cells may be compensating for the loss of HLA on antigen presentation cells [49, 50].

The SW480 and SW620 cell lines are routinely categorised as colorectal adenocarcinoma [51]. There were minor differences in the effects of SW480 and SW620 cell lines, which are models of primary versus metastatic lesions respectively. While both inhibited three DC markers, SW480 inhibited CD80 and SW620 inhibited CD54. This may suggest that, in addition to the type and localization of the primary GI tumour, but also the nature of the malignant lesion could potentially have a distinct effect on DC function in the TME. Using ex vivo TCM, generated using tumour biopsy explants from oesophageal, rectal and colonic adenocarcinoma, we assessed their differential effects on LPS-induced DC maturation. For DC surface markers in the LPS-stimulated setting, oesophageal cancer enhanced five DC markers (CD54, CD80, HLA-DR, CD86 and CD83), rectal cancer enhanced the levels of three DC markers (CD80, CD86 and CD83), whereas colonic cancer inhibited the levels of five DC markers (CD54, HLA-DR, CD86, CD83 and PD-L1) compared to LPS-induced levels in respective background media. Saying that, there was inter-individual variability apparent in the DC maturational capacity induced by ex vivo TCMs from the same GI tract tumour type. In this study, we confirmed the previously reported inhibition of DC markers by colonic adenocarcinoma, and we identified that LPS-induced PD-L1, which has a key role in the resolution of inflammation as the ligand for PD-1, is also inhibited [18–20, 52]. While the reason is unclear, we speculate that this highlights the poor potential of DCs to respond to maturational stimuli in any capacity in the colonic cancer setting. Immunophenotyping tumour-infiltrating DCs across GI cancers would be important to confirm if this effect occurs in vivo, although quantification of maturation markers, including CD83, can be difficult [53]. In our future studies, additional DC membrane markers will be included such as TLRs and other innate receptors in order to better understand the DC priming phenotypes induced by TCM. While functionally mature DCs are required in order to enable antigen presentation and T cell clonal expansion, co-culturing TME-conditioned DCs with T cells would further elucidate the overall functional significance in future studies [20]. Different types of immunosuppressive dendritic cells have been found in cancer patients and animals [54]. Lutz et al., 2002 proposed that tolerance occurs with either partial- or semi-maturation of DCs, whereas only full DC maturation is immunogenic and the decisive signal is the release of proinflammatory cytokines from DCs [55].

Interestingly, in vitro conditioned media from both OAC and CRC lines inhibited LPS-induced levels of TNF-α in DC secretions, but had no significant effect on IL-12p70. Similarly, ex vivo TCM from all three GI cancer types significantly inhibited LPS-induced levels of TNF-α in DC secretions. There were differential effects on LPS-induced levels of IL-12p70 in DC supernatants based on adenocarcinoma type with oesophageal having no effect, rectal significantly enhancing, and colonic significantly reducing levels compared to LPS in corresponding background media alone. Thus, we confirmed the known IL-12p70 inhibition and identified that DC TNF-α is also significantly inhibited by the colonic TME [18, 20]. As TNF-α from DCs is considered immunostimulatory, this then supports an extensive immunosuppressive phenotype induced by the colonic TME, in particular as shown by DC IL-12p70 inhibition, a requirement for optimal anti-tumour immunity [24, 26]. There is a self-regulatory feedback on DCs of IL-12 and TNF-α, which may occur in order to limit the effects of DCs [26]. The notable limitations of this study are the use of a single PBMC donor to derive DCs for each experiment, which was performed to confine inter-individual variability to cancer donors, and the limited number of  treatment-naïve human tumour biopsies (*n* = 8–14). While these need to be addressed to confirm reproducibility of our observations, the finding of TNF-α inhibition occurred across all cancer types and using both in vitro and ex vivo cancer samples. As the finding was common to both in vitro and ex vivo models, this indicates that soluble factors from the tumour epithelial cells may underlie this inhibition, rather than non-epithelial cells of the tumour microenvironment. Our finding of common TNF-α inhibition by GI cancers may have implications for TNF-α blockade, as has been proposed to overcome resistance to anti-PD-1 treatment [56]. Bertrand et al., 2017 proposed that TNF deficiency may favour DC accumulation in tumours, while reducing the expression of PD-1 ligands [56]. In line with this, our findings of reduced TNF-α could potentially indicate accumulation in the GI tract tumour setting of DCs with altered maturational capacities. We have previously shown that the TME of both early- and late-stage colonic cancer is equally suppressive for maturational capacity in DCs [41]. Interestingly, Scarlett et al., 2012 demonstrated that tumour-resident DCs are transformed from immunostimulatory to immunosuppressive during tumour progression in a mouse model of ovarian cancer [57]. Thus if DCs have a dynamic, even immunosuppressive, function in cancer as suggested by our study, then one could speculate from our findings that activation of suboptimally matured DCs in oesophageal adenocarcinoma could potentially result in a poorer outcome than in a setting where DC maturation is more completely suppressed, such as in colonic adenocarcinoma. When considering the implications for the findings here, it should be noted that this study is based on a model reflecting only a part of a complex system, specifically it is a model of the humoral components of the TME. These findings support conducting a larger study to determine if a negative correlation exists with outcomes, such as radioresponse and 5-year survival, across GI cancers. A larger study is also required to address some important experimental limitations of this study – in particular that DCs from multiple healthy donors should be employed, additional treatment-naïve tumour samples and non-cancerous matching GI tissues and /or cells as controls.

In this study, 2Gy-irradiation of cell lines and tumour explants was performed to correspond with the physiological effects of radiotherapy treatment at the tissue level as is performed clinically for oesophageal and rectal adenocarcinoma [42]. We found an inhibitory effect of irradiation on DC maturation through conditioned media from both in vitro and ex vivo models. Whether this finding, that TCM from 2Gy-irradiated TME inhibited DC maturation, has relevance to tumour response to radiotherapy warrants further investigation as mentioned above. However, no significant effect was observed with the clinical outcome of tumour regression grade on this small oesophageal and rectal adenocarcinoma patient cohort in this study (*n* = 14 and 10 respectively, data not shown). We propose that irradiation of the TME alters release of unknown soluble factors that are discernible to DCs, which fits with a mechanism of the radiation-induced bystander effect via altered levels of inflammatory cytokines produced by the TME [58–62]. However, the irradiated TCMs had no effect on DC secretion levels of TNF-α or IL-12p70, therefore the functional significance of the effect on DC markers is difficult to decipher in terms of the ability of DCs to potentiate any immunomodulatory message to other bystander cells. Inflammation and angiogenesis are closely related and may underlie immune inhibition and radioresponse [20]. Unfortunately, profiling the ex vivo TCM for inflammatory and angiogenic factors did not identify differences in levels of any of the mediators between 0Gy- and 2Gy-irradiated patient-matched biopsies. Radiotherapy has been described as both immunostimulatory and immunosuppressive with radiation dose proposed to be a key influencer in this, where low dose radiation, such as 2Gy, may be immunosuppressive [32, 37, 63]. This data could suggest that radiotherapy of the GI TME may further reduce DC maturation, which we speculate could be beneficial in improving outcome in a setting where DCs may only have the capacity to sub-optimally mature, such as oesophageal adenocarcinoma.

As tumour biopsies contain tumour epithelial cells in addition to other cell types, ex vivo TCM contains many different tumour associated soluble factors, and therefore closely mimics the inflammatory milieu of the tumour in situ. Several cytokines and chemokines have been described to be present at high levels in the colonic TME compared to normal tissues, such as CXCL1 and CXCL5 (which function to attract and activate neutrophils) and CCL2 (a chemoattractant for monocytes, memory T cells and DCs) [20]. We do not yet know the mechanistic pathways or secreted factors that induce the DC phenotypes we observed. Although NF-κB would be a candidate pathway given its reported roles in both LPS-induced DC maturation that ultimately results in the activation of NF-κB and the production of proinflammatory cytokines and in radiation-triggered TNF-α - NF-κB cross-signalling [62, 64, 65]. In this study, levels of specific inflammatory and angiogenic mediators in ex vivo TCM of oesophageal, rectal and colonic adenocarcinoma were correlated with DC maturation marker - CD54. Thus it is possible that low levels of the cytokine IL-2 and high levels of angiogenic mediators - Ang-2 and bFGF, in TCM of tumour biopsies may confer a more DC inhibitory environment and this fits with some expected roles for these mediators. As CD54 (also known as intercellular adhesion molecule 1, ICAM-1) promotes DC-T cell binding, this indicates possible negative effects on the capacity of DCs to activate T cells in order to induce an adaptive immune response in GI tumours with lower levels of IL-2 and higher levels of Ang-2 and bFGF. IL-2 has key functions in the immune system, tolerance and immunity, primarily via direct effects on T cells, both effector and regulatory type. Interestingly, a role has been proposed for DC maturation as a mediator of systemic IL-2 effects [66]. DCs can differentiate into endothelial-like cells when cultured in the presence of angiogenic growth factors – bFGF, VEGF and IGF-1, and these altered DCs have a reduced functional potency [67]. We have previously shown that levels of tumour vasculature maturity or DC inhibition negatively correlate with survival of colonic adenocarcinoma patients on anti-angiogenic treatment [40, 68]. We have previously identified that multiple mediators influence DC inhibition in colonic TCM - increasing levels of CXCL1, CXCL5, CCL2 and VEGF in the TCM correlated with inhibition of IL-12p70 secretion from DCs, however these isolated factors were not sufficient to induce all aspects of the extensive DC inhibition as observed for colonic TCM [20]. The data from this study further supports the concept that it may be the cumulative effect of many mediators in the TME that may influence DCs. In particular, the levels of TGF-beta may be relevant due to its pivotal role in inducing immunological tolerance in DCs in colonic tissue [69, 70]. While the effects of distinct ex vivo TCMs on DCs are described relative to their corresponding background media, profiling the levels of mediators may not be representative of the in vivo setting due to differences in culturing conditions. In future studies, healthy tissue controls and cells will be examined in conjunction with cancerous samples. GI cancers are well known to be molecularly heterogeneous with, for example, a substantial proportion of tumours (15% of CRC) displaying microsatellite instability phenotype and these tumours are commonly highly immunogenic, densely infiltrated with activated T cells and respond well to immune checkpoint blockade therapy [71]. Therefore it would be interesting to examine if this subset of CRC tumours are not able to inhibit DC maturation to the same extent as observed in this study for which we do not know the MSI phenotype of the tumour biopsies.

## Conclusions

**Fig. 5:**
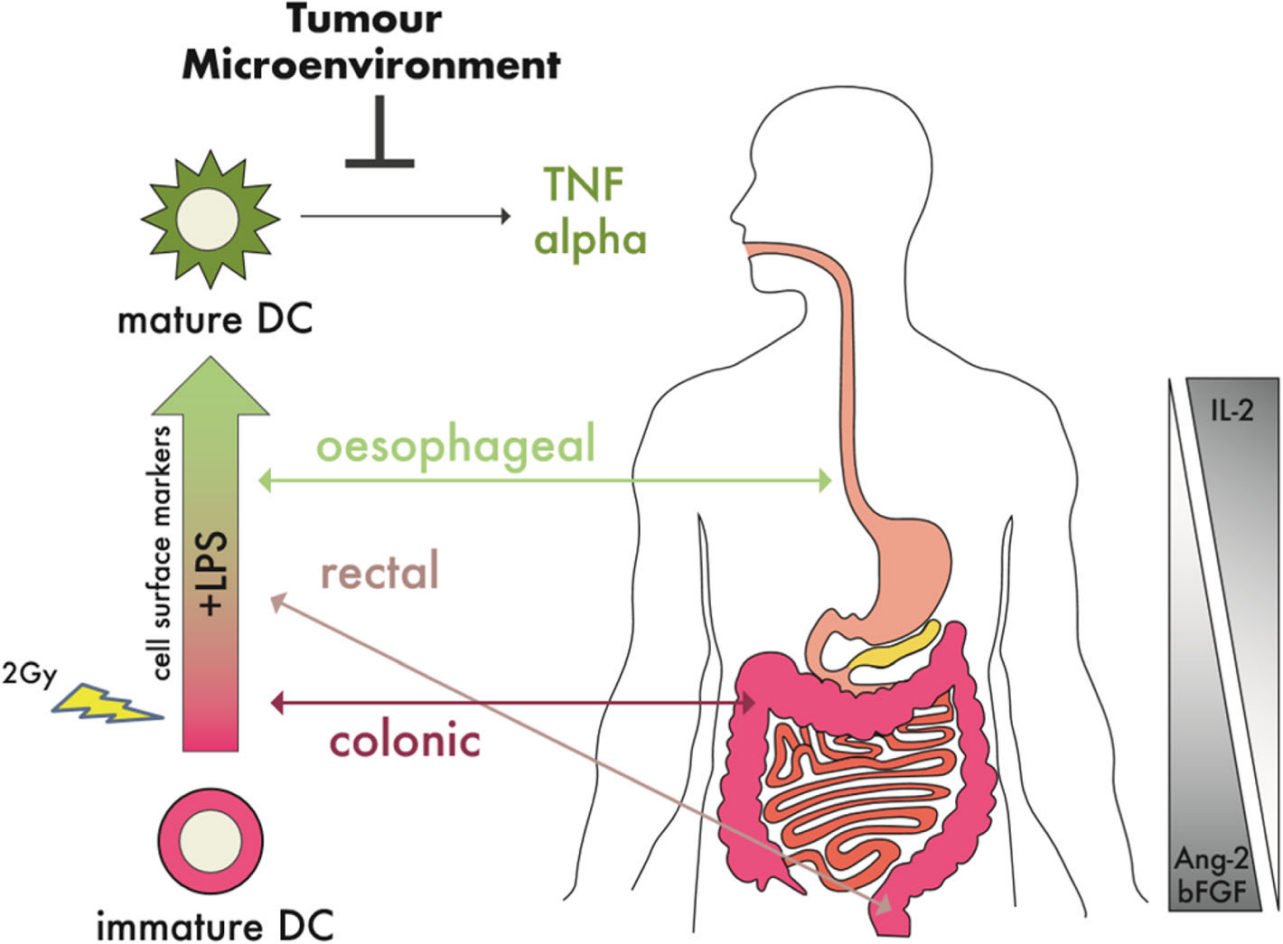
While there was varying effects on DC maturational capacity induced by distinct gastrointestinal adenocarcinoma types, oesophageal, rectal and colonic TME inhibited DC secreted TNF-α. This model summarises that treatment of DCs with GI TCM revealed differential effects on DC maturational capacity with oesophageal cancer enhancing most markers, rectal cancer enhancing three markers and with colonic cancer inhibiting most markers. In addition, 2Gy-irradiation of the TME inhibited LPS-induced levels of DC markers. Differential levels of angiogenic and inflammatory mediators in ex vivo TMEs correlated with effects on DC surface markers, with IL-2 positively correlating and with Ang2 and bFGF negatively correlating with the DC maturation marker CD54. Regardless of the effect on DC surface markers, the TME of all GI tract cancer types significantly inhibited DC secreted TNF-α levels

In conclusion, this study demonstrates for the first time that there are differences in the levels of DC maturational capacity across distinct human GI adenocarcinomas (Fig. 5). The consistent and significant inhibition of DC TNF-α across oesophageal, rectal and colonic adenocarcinoma may be the key way in which DC maturation is dysregulated by GI cancers. In addition, radiotherapy-mimicked TCM further inhibited DC maturational capacity at the maturation marker level. Differential levels of secreted mediators of inflammation (IL-2) and angiogenesis (Ang2 and bFGF) in the TME may underlie variation in DC responsiveness. This in turn may reduce the capacity of localised DCs to induce an anti-tumour immune response and may have implications for response to radiotherapy. As the oesophageal TME appears to be less inhibitory compared to colonic adenocarcinoma, this may have implications for informing immunotherapy, such as DC vaccines, and PD-1/PD-L1 or TNF-α blockade [56, 72, 73]. Indeed, LPS-induced levels of PD-L1 on TME-conditioned DCs was only inhibited by colonic adenocarcinoma, but not oesophageal or rectal adenocarcinoma. This study warrants further functional and in vivo investigations as it may have implications for how localised DCs are inhibited from inducing anti-tumour immunity in GI cancer and may also influence response to radiotherapy.

## Supplementary information

**Additional file 1 Supplementary Table 1**. Patient demographics. **Supplementary Fig. 1**. Experimental outline and flow cytometry gating strategy and staining controls. **Supplementary Fig. 2**. ex vivo TCM from distinct gastrointestinal adenocarcinoma types induced differential effects on LPS-induced DC maturation. **Supplementary Fig. 3**. ex vivo TCM from distinct gastrointestinal adenocarcinoma types induced differential effects on unstimulated DC marker levels. **Supplementary Fig. 4**. in vitro TCM of 2Gy-irradiated cell lines from gastrointestinal cancers induced significant inhibition of DC markers compared to mock irradiation. **Supplementary Fig. 5**. ex vivo TCM of 2Gy-irradiated TME from gastrointestinal cancers inhibited DC markers compared to mock irradiation

## Data Availability

The datasets used and/or analysed during the current study are available from the corresponding author on reasonable request.

## Abbreviations

CD: Cluster of Differentiation
cM199: Complete Medium 199
CRC: Colorectal Cancer
cRPMI: Complete Roswell Park Memorial Institute 1640 Medium
CRT: Chemoradiotherapy
DC: Dendritic Cell
ELISA: Enzyme-Linked Immunosorbent Assay
FMO: Fluorescence Minus One
GI: Gastrointestinal
Gy: Gray
HLA-DR: Major Histocompatibility Complex, Class II, DR
IL: Interleukin
LPS: Lipopolysaccharides
OAC: Oesophageal Adenocarcinoma
PBMCs: Peripheral Blood Mononuclear Cells
PD-L1: Programmed Cell Death 1-Ligand 1
TCM: Tumour Conditioned Media
TME: Tumour Microenvironment
TNF-α: Tumor Necrosis Factor-Alpha
