# Supplementary material for: The tumour microenvironment of the upper and lower gastrointestinal tract differentially influences dendritic cell maturation
Source: BMC Cancer. 2020 Jun 17;20:566. doi: 10.1186/s12885-020-07012-y (PMC7302160; doi:10.1186/s12885-020-07012-y)
Supplement: Supplementary file 1 — Additional file 1 Supplementary Table 1. Patient demographics. Supplementary Fig. 1. Experimental outline and flow cytometry gating strategy and staining controls. Supplementary Fig. 2. ex vivo TCM from distinct gastrointestinal adenocarcinoma types induced differential effects on LPS-induced DC maturation. Supplementary Fig. 3. ex vivo TCM from distinct gastrointestinal adenocarcinoma types induced differential effects on unstimulated DC marker levels. Supplementary Fig. 4. in vitro TCM of 2Gy-irradiated cell lines from gastrointestinal cancers induced significant inhibition of DC markers compared to mock irradiation. Supplementary Fig. 5. ex vivo TCM of 2Gy-irradiated TME from gastrointestinal cancers inhibited DC markers compared to mock irradiation [file 12885_2020_7012_MOESM1_ESM.docx]

Supplementary

**Supplementary Table 1. Patient demographics**

| **Gastrointestinal Adenocarcinoma** | | | | | |
| --- | --- | --- | --- | --- | --- |
| **Type** | **Oesophageal** | | **Rectal** | **Colonic** | |
| **Collection Dates** | 01 2013 - 06 2013 | 06 2013 - 09 2014 | | 01 2014 - 09 2015 | |
| n | 14 | | 10 |  | 8 |
| **Gender** |  | |  |  |  |
| Male | 12 | | 8 |  | 5 |
| Female | 2 | | 2 |  | 3 |
| **Age at Diagnosis (years)** |  | |  |  |  |
| Average | 62 | | 68 |  | 70 |
| Range | 39-76 | | 60-82 |  | 45-81 |
| **TNM Stage** |  | |  |  |  |
| T Stage |  | |  |  |  |
| T2 . | 2 | | 4 |  | 2 |
| T3 . | 12 | | 6 |  | 5 |
| T4 . |  | |  |  | 1 |
| N Stage |  | |  |  |  |
| N0 . | 6 | | 2 |  | 2 |
| N1 . | 2 | | 5 |  | 3 |
| N2 . | 4 | | 3 |  | 3 |
| N3 . | 2 | |  |  |  |
| M Stage |  | |  |  |  |
| M0 . | 11 | | 7 |  | 7 |
| M1 . | 2 | | 3 |  | 1 |
| Mx . | 1 | |  |  |  |
| **Differentiation or**  **Dukes' Stage** | Differentiation | | Differentiation |  | Dukes' Stage |
| Poor | 5 | |  | A | 1 |
| Moderate | 7 | | 9 | B | 1 |
| Well | 2 | |  | C | 4 |
| n/a |  | | 1 | n/a | 2 |

Characteristics of the cohort of patients with gastrointestinal adenocarcinomas that provided samples for this study (n=32). The values indicate the collection dates (month year), number of patients and age in years as appropriate.


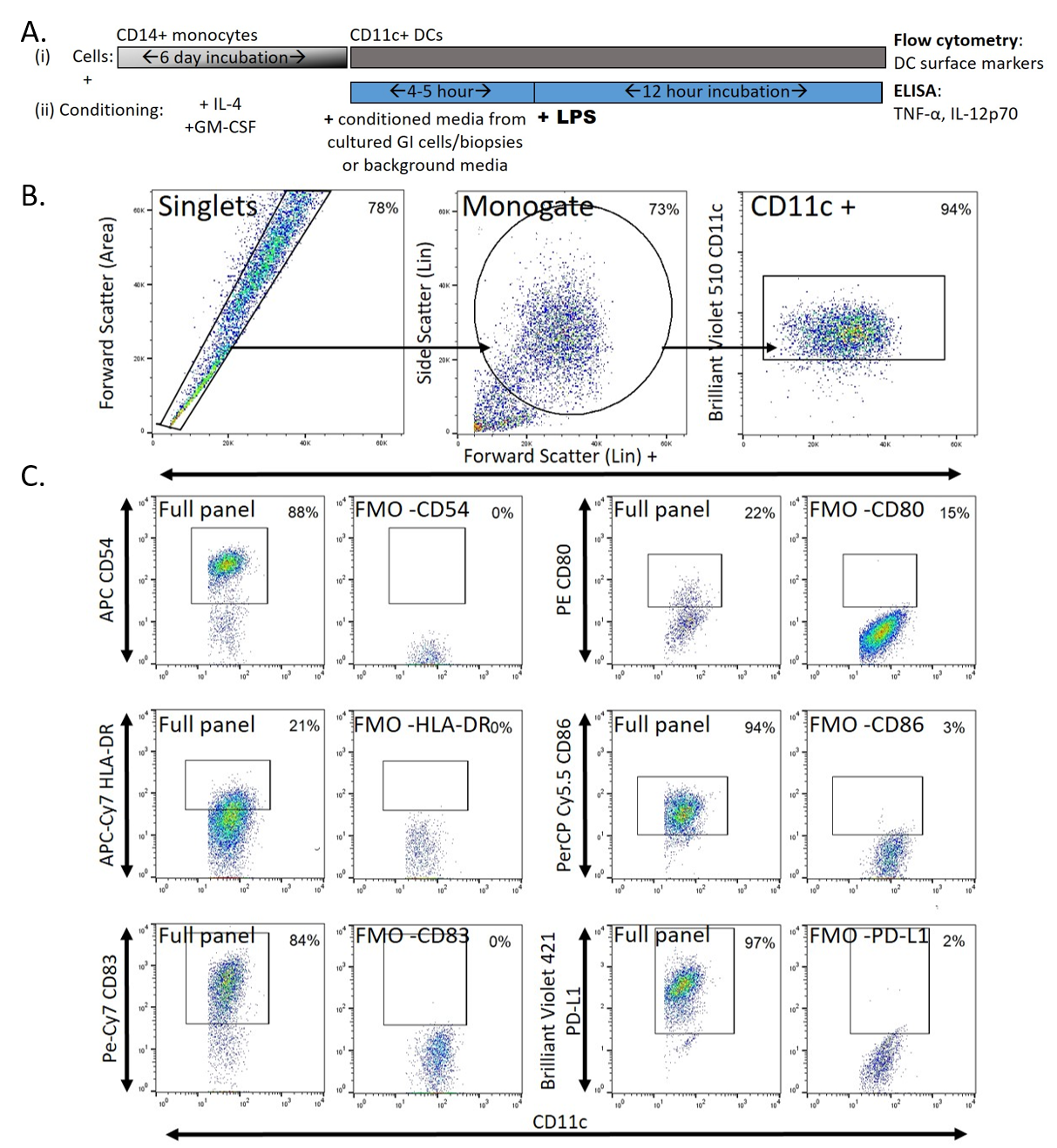
**Supplementary Fig. 1. Experimental outline and flow cytometry gating strategy and staining controls.**

**A** The experimental outline is illustrated describing the incubation of (i) DC preparations for the specified time and (ii) the conditions to which the cells were exposed, specifically IL-4 and GM-CSF cytokines to derive the DCs, conditioned media from the specified gastrointestinal sources to precondition the DCs and LPS to mature the DCs. Finally, DCs were analysed by flow cytometry and DC supernatants were analysed by ELISA. **B-C** The gating strategy of the monocyte-derived DCs is shown of singlet, monogate cells which are CD11c+ (B) and the Fluorescence Minus One staining controls (C).

**Supplementary Fig. 2. *ex vivo* TCM from distinct gastrointestinal adenocarcinoma types induced differential effects on LPS-induced DC maturation**

**
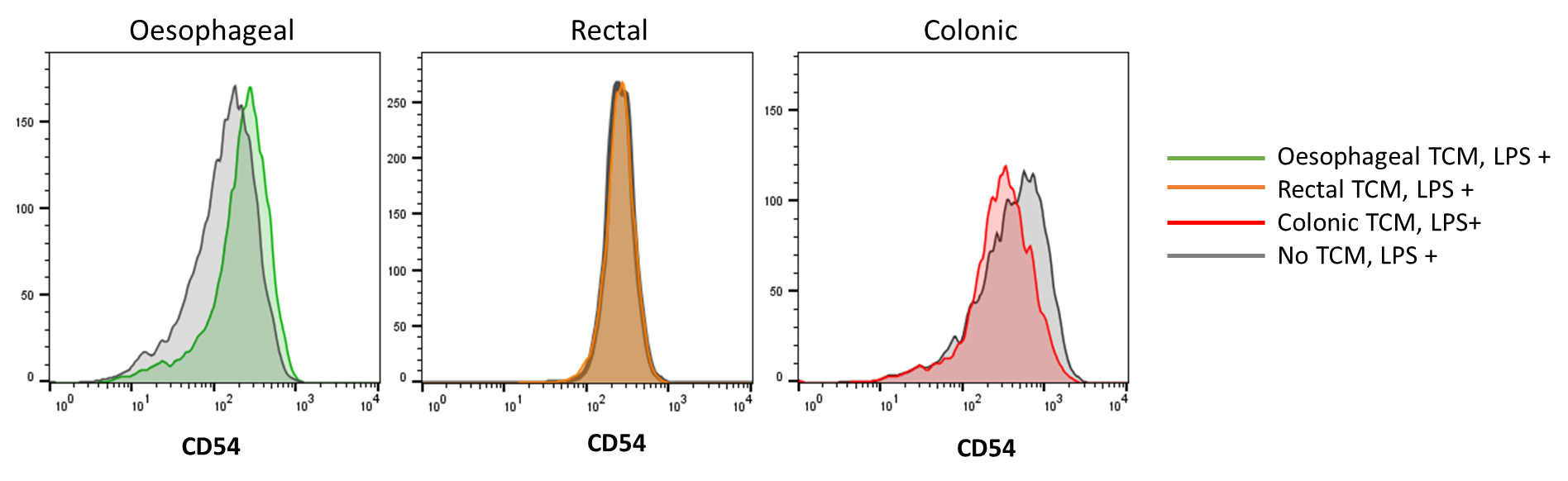
**

Representative histograms of LPS-induced levels of DC maturation marker CD54 when treated with oesophageal, rectal or colonic TCM compared to background media.

**Supplementary Fig. 3. *ex vivo* TCM from distinct gastrointestinal adenocarcinoma types induced differential effects on unstimulated DC marker levels**


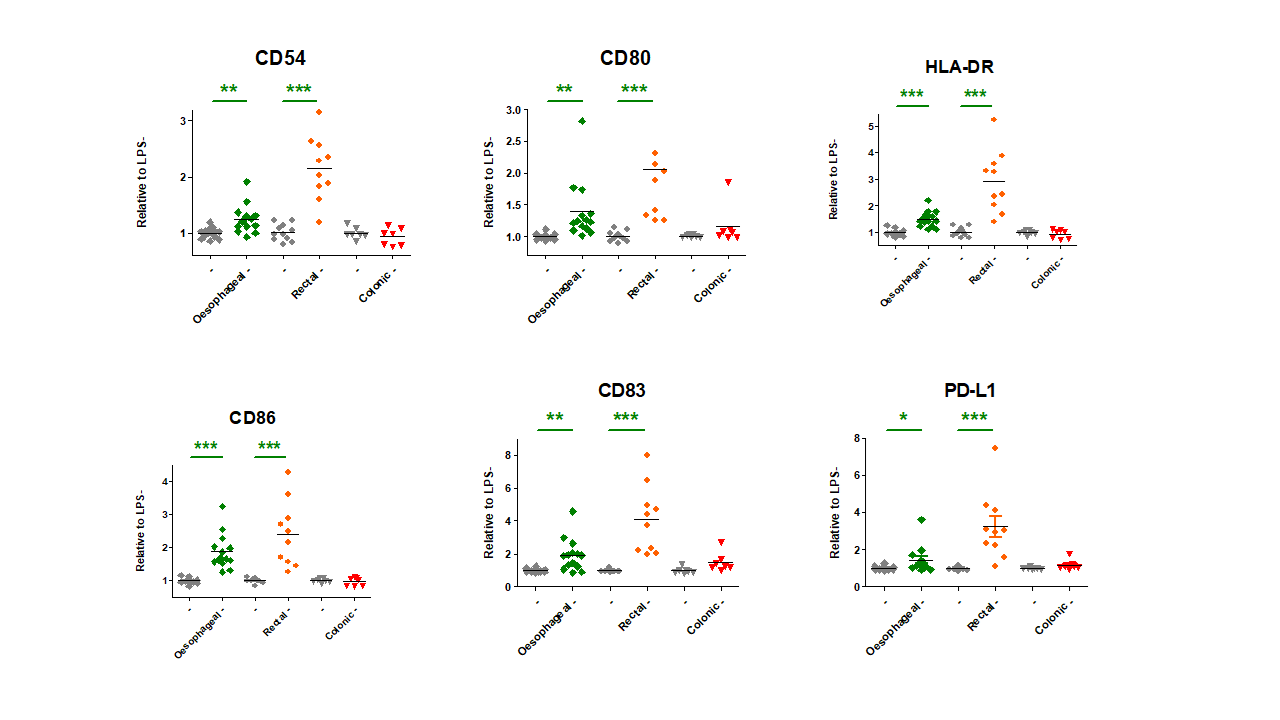


While both oesophageal TME (green squares, n=14) and rectal TME (orange circles, n=10) significantly induced all markers, the colonic TME (red triangles, n=7) showed no activation over unstimulated levels (-, where grey diamonds, circles and triangles indicate unstimulated levels in appropriate background media for oesophageal, rectal and colonic adenocarcinoma TCM respectively). Data is from DC preparations generated from one healthy PBMC donor per cancer type. Statistically significant (unpaired t-test) modulation of DC maturation relative to LPS control (+, grey bars to the left of each cancer type) is indicated by asterisks, where green asterisks indicate significant induction and red asterisks indicate significant inhibition and p≤0.050 is *, ≤0.005 is ** and ≤0.0005 is ***.

**Supplementary Fig. 4. *in vitro* TCM of 2Gy-irradiated cell lines from gastrointestinal cancers induced significant inhibition of DC markers compared to mock irradiation**


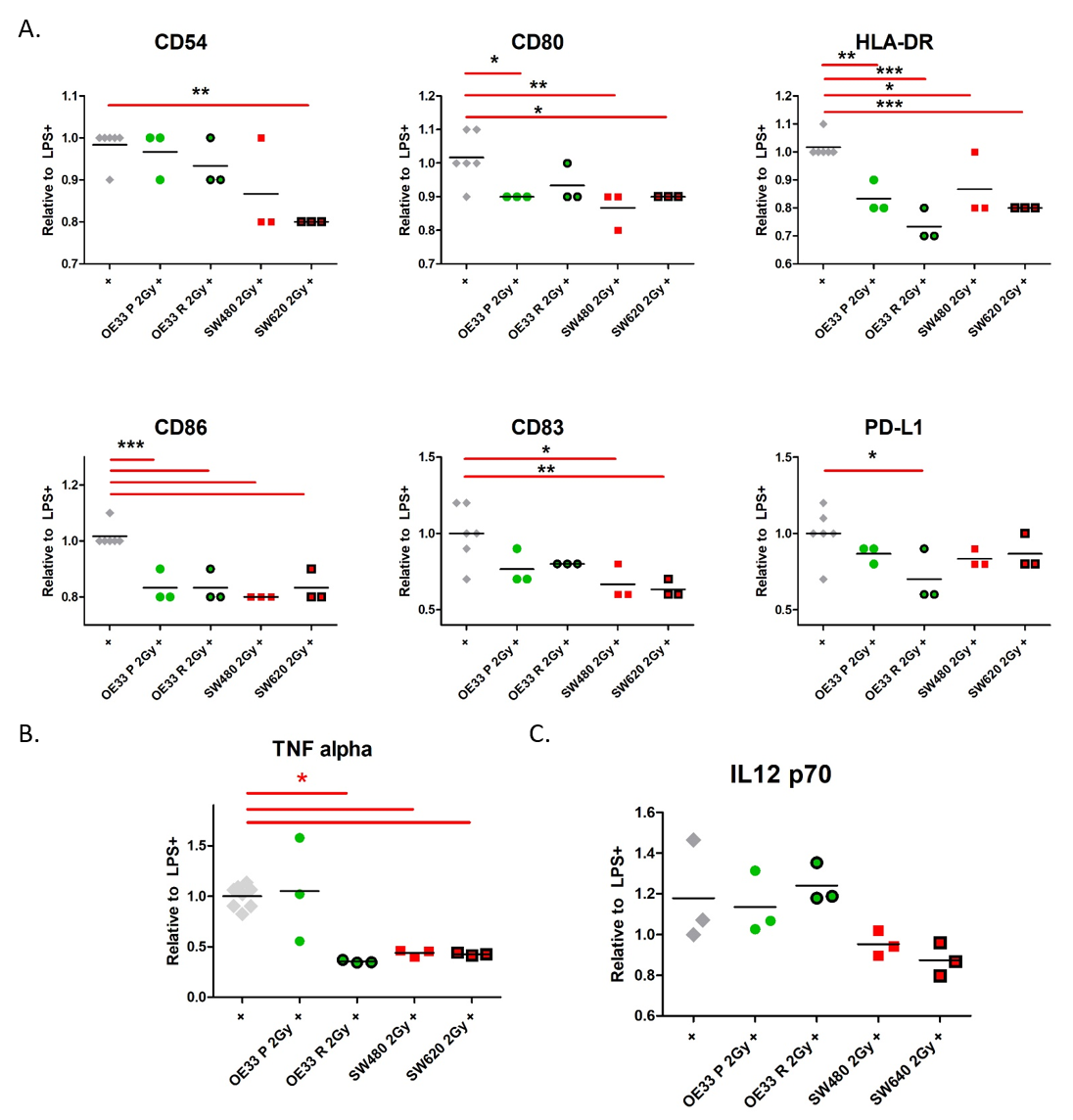


(A) Inhibition of DC markers CD80, HLA-DR and PD-L1 was induced by at least one of the 2Gy-irradiated OAC cell lines relative to LPS-activated levels (+, grey diamonds). Inhibition of DC markers CD54, CD80, HLA-DR, CD86 and CD83 was induced by at least one of the 2Gy-irradiated CRC cell lines relative to LPS-activated levels (+, grey diamonds). (B-C) Cytokine levels in TME-conditioned DC supernatants showed significant inhibition of LPS-activated levels of TNF-α by 2Gy-irradiated OE33 R, SW480 and SW620 cell lines (B), though no significant differences were observed for IL12 p70 (C). DC maturation levels are shown relative to LPS-activated maturation (+, grey diamonds) and statistical comparison of *in vitro* TCM+LPS (n=3) from 2Gy-irradiated cell lines is performed relative to LPS-activated maturation alone (+, n=3-6). LPS-activated levels of DC markers as influenced by treatment with OAC lines, OE33 Parental and OE33 Radioresistant, is shown in green circles and by CRC lines SW480 and SW620 is shown in red squares. Data is from one DC preparation generated from one healthy PBMC donor. Statistically significant (ANOVA with Dunnett's Multiple Comparison Test) inhibition of DC maturation relative to the LPS-activated levels (+, grey diamonds) is indicated by asterisks.

**Supplementary Fig. 5. *ex vivo* TCM of 2Gy-irradiated TME from gastrointestinal cancers inhibited DC markers compared to mock irradiation**


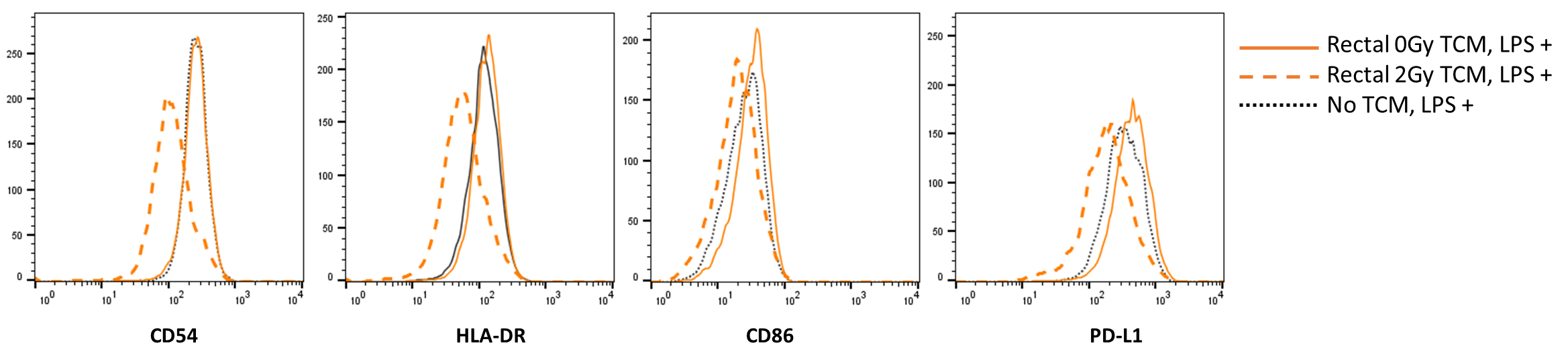


Representative histograms of LPS-induced levels of DC maturation markers CD54, HLA-DR, CD86 and PD-L1 when treated with rectal TCM from 0Gy- (solid orange line) or 2Gy- (dashed orange line) irradiated biopsies, or background media (dashed black line).
